# Synthesis of Extended Atomically Perfect Zigzag Graphene - Boron Nitride Interfaces

**DOI:** 10.1038/srep16741

**Published:** 2015-11-20

**Authors:** Robert Drost, Shawulienu Kezilebieke, Mikko M. Ervasti, Sampsa K. Hämäläinen, Fabian Schulz, Ari Harju, Peter Liljeroth

**Affiliations:** 1 Department of Applied Physics, Aalto University School of Science, PO Box 15100, 00076 Aalto, Finland; 2 COMP Centre of Excellence, Department of Applied Physics, Aalto University School of Science, PO Box 11100, 00076 Aalto, Finland

## Abstract

The combination of several materials into heterostructures is a powerful method for controlling material properties. The integration of graphene (G) with hexagonal boron nitride (BN) in particular has been heralded as a way to engineer the graphene band structure and implement spin- and valleytronics in 2D materials. Despite recent efforts, fabrication methods for well-defined G-BN structures on a large scale are still lacking. We report on a new method for producing atomically well-defined G-BN structures on an unprecedented length scale by exploiting the interaction of G and BN edges with a Ni(111) surface as well as each other.

With the ever-growing family of two dimensional (2D) materials, now comprised of the semi-metal graphene (G)^1^,^2^, insulators such as hexagonal boron nitride^3^ (BN), and semiconductors such as the transition metal dichalcogenides^4^ and phosphorene^5^,^6^, new opportunities arise from the combination of these different materials into heterostructures^7^. Recent experiments have shown the potential of out-of-plane stacked structures of 2D materials for assembling transistor structures reminiscent of those currently used in the semiconductor industry^7^,^8^,^9^, while the combination of 2D materials covalently bonded together in the same plane promises to give control over their electronic properties and yield materials with new emerging properties. In particular, the combination of graphene with BN has received attention and many exciting properties such as magnetism and half-metallicity have been predicted theoretically^10^,^11^,^12^,^13^,^14^,^15^. While fabrication techniques for graphene-BN (G-BN) heterolayers with accuracies down to 100 nm have been demonstrated^16^,^17^, the ultimate goal lies in the atomic scale control of the interface between the two materials as atomically precise graphene nanostructures have been proposed for important graphene applications. Zigzag (ZZ) terminated nanostructures could enable spintronics in graphene^18^,^19^ and help to realise phenomena in graphene for which no analogue exists in silicon technology, such as valleytronics^20^,^21^. Passivation of such nanostructures with BN would allow their transfer onto different substrates without altering the atomic structure that is crucial for their functionality.

Two main approaches have been used to fabricate G-BN heterostructures thus far: The pattern re-growth technique uses photolithographic patterning of graphene or BN layers, generally grown on metal foils^16^,^22^. The holes in the pattern are then filled up with the other material by chemical vapor deposition (CVD) growth. Shape control down to 100 nm has been demonstrated in the pattern re-growth approach. This technique is capable of producing both intricate and large scale G-BN patterns. However, there is no atomic scale control of the G-BN interface and, in general, no registry exists between the graphene and BN lattices. A more promising approach for interface control is the epitaxial, sequential growth of graphene and BN on transition metal single crystals, where regular edge structures are readily obtained. In these cases, the edge of the first material will act as a seed for the growth of the second, leading to a shared lattice orientation^17^,^23^,^24^,^25^,^26^.

Experiments in the past few years have focused on well-established surfaces for graphene growth such as Ir(111)^25^,^27^, Rh(111)^28^, Ru(0001)^17^,^23^,^26^ and copper foils^24^. Common to all of these surfaces is the formation of a moiré pattern in both graphene and BN regions owing to the mismatch between the surface and adlayer lattice constants. Even though graphene and BN have very similar lattice constants, their moiré patterns show far larger differences^29^,^30^,^31^, leading to strain-induced defect formation close to the interface on at least some of these surfaces^26^.

Ni(111) is a strongly interacting crystal facet with a lattice constant closely matched to that of both graphene and BN. As a result of the strong interaction, both materials form 1 × 1 structures on Ni(111)^32^,^33^,^34^,^35^,^36^. The absence of a moiré superstructure on Ni(111) makes it an ideal surface for the controlled synthesis of extended, atomically perfect interfaces of graphene and BN. We study the formation of interfaces between graphene and BN grown under ultra-high vacuum (UHV) conditions on Ni(111) by low temperature scanning tunneling microscopy (STM) and show that extended, high quality interfaces are readily obtained on this surface by using epitaxial BN islands as seeds for graphene growth.

## Results

**Figure 1:**
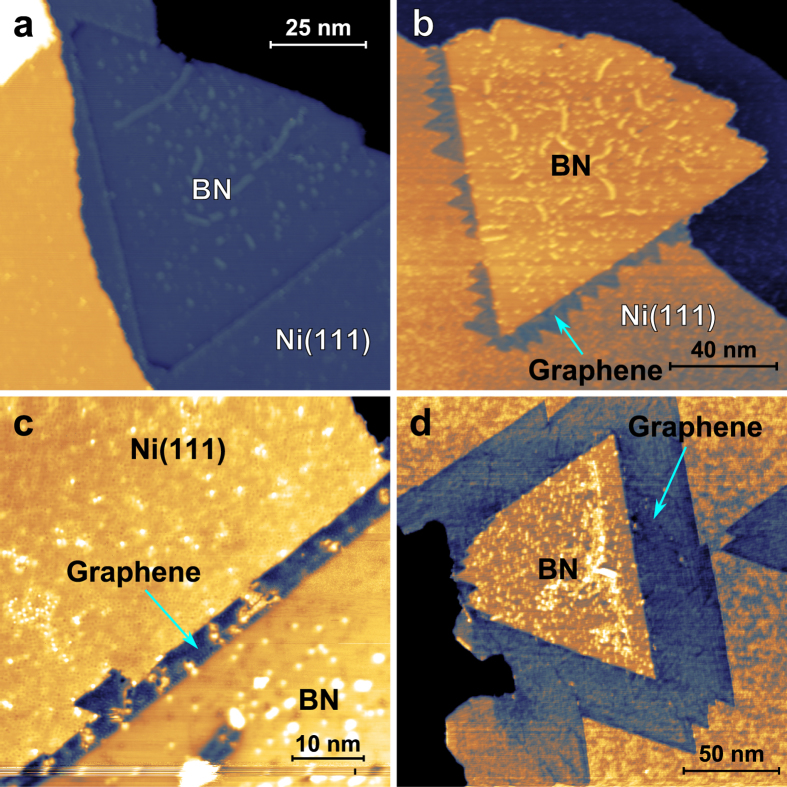
Sample at different stages of growth. (**a**) A triangular BN island as grown on Ni(111) by introducing 3.2 Langmuir (L) of borazine at 1120 K and 2 × 10^−8^ mbar with annealing for 20 minutes (0.1 V, 1 nA). (**b**) Partially embedded G-BN domain grown by exposure of the BN sample to 16 L ethylene at 830 K and 10^−7^ mbar with rapid seeding, resulting in triangular graphene domains (see experimental section for details). Free BN edges uncovered with graphene are seen in the top of the island (2 V, 100 pA). (**c**) G-BN sample after 16 L of exposure to ethylene at 830 K and 10^−7^ mbar. At lower pressure, the growth front propagates parallel to the BN edge, producing narrow graphene ribbons (0.1 V, 500 pA). (**d**) G-BN sample after 48 L of exposure to ethylene at 830 K and 10^−7^ mbar. Graphene growth proceeds parallel to the BN edge (1 V, 100 pA).

Figure 1 shows the sample at different stages of the preparation process. BN grown by low pressure CVD in the 10^−8^ mbar range displaces nickel atoms from Ni(111) terraces to form highly symmetric triangular islands embedded into the first metal layer. This growth mode has also been observed for graphene on the Ni(111) surface^35^. While the few free BN edges exhibit some roughness, the BN/Ni(111) interface of embedded domains is straight at the atomic level, see Fig. 1a and Figure S1 in the supporting information. The edges of these embedded BN triangles serve as seeds for the nucleation of graphene (Fig. 1b), which continues to displace nickel surface atoms in the second growth step, while the nucleation of graphene on the bare Ni(111) or on free BN edges is only rarely observed. This replacement of surface atoms is typical for graphene growth on Ni(111) in the absence of carbon impurities^35^. Graphene and BN are easily distinguishable as they appear with different contrast in the STM and an initial assignment can be made with knowledge of the growth sequence, relative abundance, work function measurements (see supporting information), and the appearance of the BN triangles before graphene is attached. Depending on the imaging parameters used, the apparent height difference between graphene and BN can be up to 100 pm, much smaller than the Ni(111) step height of 173 pm^37^. At low bias, the two 2D crystals are clearly shown to be in the same plane and embedded into the first atomic layer of the Ni(111) surface.

Shape control of the graphene domains attached to the BN seeds may be achieved by controlling the ethylene pressure during the growth process. Sharp triangular domains emerging from the BN seed are obtained if the ethylene pressure is high in the first few seconds of the growth. We speculate that graphene then nucleates simultaneously at several points of the BN edge and these domains merge to the jagged structures seen in Fig. 1b as the individual grains expand at reduced pressure (see also Figure S1 in the supporting information). Low pressure CVD (10^−7^ mbar of ethylene or less) on the other hand results in a growth front proceeding parallel to the ZZ edge of the BN seed, forming narrow ZZ terminated graphene strips. The width of these strips can be tuned through the deposition time. By carefully controlling the preparation conditions, we have produced well-defined graphene strips between 4 nm (Fig. 1c) and 34 nm (Fig. 1d) in width.

The BN seeds retain their original triangular shape during graphene attachment, leading us to conclude that the BN edge is stable under reaction conditions for graphene growth. This interpretation agrees well with the fact that the temperatures needed to obtain high quality BN on the Ni(111) surface exceeds that for graphene growth by several hundred degrees (see experimental section). As a result, an atomically perfect ZZ interface between BN and graphene is readily obtained. Graphene attached to BN domains remains embedded in the surface and forms a remarkably straight interface with the Ni(111) surface along the ZZ direction (see Fig. 1 and supplemental Figure S1). This suggests that the method presented here could be adapted for producing more complicated heterostructures by attaching another material to the graphene edge.

**Figure 2:**
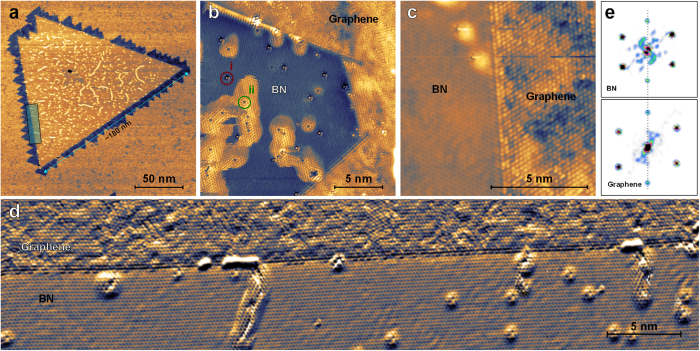
Atomic resolution images of G-BN interfaces. (**a**) BN domain lined with graphene. The area marked by a rectangle is shown in panel (d) (2 V, 100 pA). (**b**) Atomically resolved image of a G-BN structure (10 mV, 5.3 nA). All defects introduced into the BN during graphene growth are revealed to be point defects. (**c**) Atomically resolved image of a ZZ G-BN interface (10 mV, 6 nA). (**d**) Current map of an atomically resolved STM image taken on the the interface section in panel (**a**) highlighting the long-range order of the interfaces (15 mV, 3 nA). (**e**) Fourier transform of atomically resolved STM images taken on graphene and BN near an interface, highlighting the crystallographic continuity of the heterolayer.

Some defects in the BN islands are found on all samples investigated in this study. This is not of immediate concern since the functionality of G/BN nanostructures lies with the graphene areas, but a closer investigation to assess the potential impact of these defects on the G-BN interface is warranted. While the mechanism by which these imperfections are formed cannot be conclusively identified based on our experiments, the etching of the BN sheet by hydrogen abundant from the decomposition of the precursors used during the sample preparation and the substitution of B or N atoms with C are likely candidates. The defect density in BN decreases as one approaches the interface with graphene. Atomically resolved STM images of a defective area in BN, shown in Fig. 2b, further reveal that despite their large spatial extent, all defects in BN are in fact point defects. Two main types of faults with different surrounding contrast can be identified: Type (i) defects, highlighted by a dark red circle in Fig. 2b, are assumed to be substitutional in nature and do not affect the structure of BN around them. Type (ii) defects, labeled in green, are also clearly point defects, but show a halo extending several nm from the defect site. These defects are attributed to missing atoms which present a pathway for the intercalation of a foreign chemical species, most likely hydrogen, underneath the BN sheet during graphene growth. The atomic lattice on the defect halo is intact, as can be seen in Fig. 2b. We conclude that the impact of BN defects on the G-BN interface is negligible as even type (ii) defects close to the interface do not disturb the atomic lattice at the interface itself. Such atomic scale defects are not observed in the graphene areas of the sample where the dominant type of defect are small rotated domains of graphene as can be seen in Fig. 2d.

We now turn to the atomic structure of the G-BN interfaces. A close-up view of a G-BN junction in atomic resolution is shown in Fig. 2c. The interface is atomically well-defined and proceeds along the ZZ direction (note that usually only one graphene sublattice is resolved in STM images on the G/Ni(111) system). The area highlighted in the lower left hand side of Fig. 2a is shown in atomic resolution in Fig. 2d. Note that the total length of the individual G-BN interfaces in Fig. 2a exceeds 150 nm and the section shown in Fig. 2d is *ca.* 50 nm in length with only few defects. Even at these length scales, the interface is atomically sharp along the entire section shown. Finally, Fig. 2e shows the Fourier transform of atomically resolved STM images taken on the graphene and BN sides close to an interface, highlighting the continuity of the 2D crystal structure across the G-BN junction. The high quality of the BN edges and intact edge structure even after graphene attachment suggest that the total length of high quality ZZ G-BN interfaces obtained by the technique presented here is only inherently limited by the size of the BN seed employed for growth, *i.e.* only by the nucleation density of BN on the substrate.

It is clear from earlier work^32^ as well as the data shown in Figs. 1 and 2 that the equilibrium shape of BN crystals on the Ni(111) surface is a triangle, meaning that only either the B or N sublattice is exposed at the edge and only G-BN interfaces with a specific chemical connection are formed. Density functional theory (DFT) and tight-binding calculations for ZZ terminated nanoribbons embedded in BN show that the properties of G-BN structures depend heavily on the chemical nature of the G-BN interface and a preparation process that could be tailored to selectively obtain a predetermined termination would be desirable^11^,^15^,^25^. Some of the BN grains found on the surface do however not reach their equilibrium shape and remain as truncated triangles, allowing us to study the differences in C-B and C-N interface formation with the aid of detailed DFT calculations.

**Figure 3:**
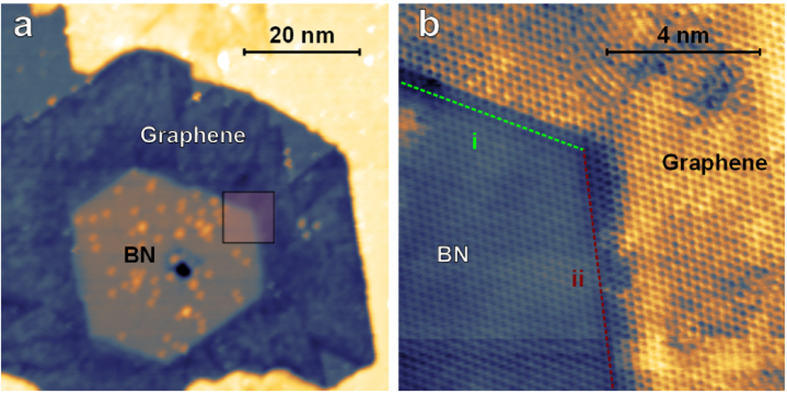
Differences between BN edges. (**a**) Truncated BN triangle encapsulated in graphene (1 V, 171 pA) (**b**) Atomically resolved STM image of a G-BN structure grown on a BN grain with B and N terminated edges. Intermixing of BN and graphene is seen on the interface in the center of the image (10 mV, 5.3 nA).

Figure 3a shows a hexagonal BN island enclosed in graphene. The two chemically distinct edges of BN are found to respond quite differently to graphene attachment, as shown in Fig. 3b. At the interface marked (i) in Fig. 3b, an atomically sharp transition along the ZZ direction is formed, similar to those reported above. No such straight junction can be made out at the interface (ii) in Fig. 3b. Instead, a *ca.* 1 nm wide transition region is observed where graphene and BN seem to intermix. This behaviour suggests that the formation of either C-B or C-N bonds is strongly preferred on the Ni(111) surface and the system attempts to minimize its energy by re-organising unfavourable interfaces.

**Figure 4:**
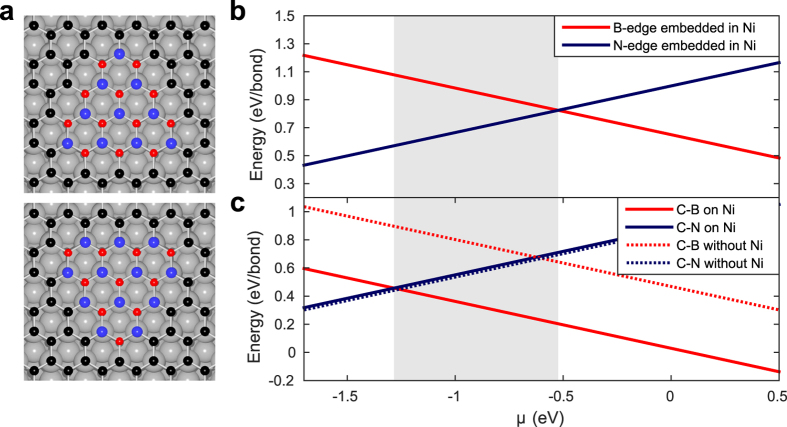
Interface energies from DFT: (**a**) Triangular h-BN nanoislands with B-terminated (top) and N-terminated (bottom) zigzag edges embedded in graphene on the Ni(111) substrate. The red, blue, black, and grey spheres represent the boron, nitrogen, carbon, and nickel atoms, respectively. (**b**) Zigzag edge energies of BN embedded in Ni(111) and (**c**) interface energies of BN embedded in graphene with or without Ni(111) substrate as a function of B atom chemical potential *μ*.

To confirm this interpretation, we performed DFT calculations (computational details are given in the numerical methods and supplementary information) to estimate the energies of the C-B and C-N interfaces on Ni(111). The previous DFT work by Huda *et al.*^38^ found that the lowest energy of BN on Ni(111) is achieved when the N atoms occupy the top sites and the B atoms are on the fcc sites, respectively. As the same sites are favoured by graphene^39^, the continuity of the lattice at the interface is ensured. It follows that the substrate constrains the relative alignment of possible interfaces, such that the orientation of a triangular BN nanoisland determines whether its zigzag edge is N or B terminated, see Fig. 4a. Moreover, the distances of BN and graphene from the Ni(111) substrate are closely matched at around 2.0 Å, the exact value depending on the experiment^40^,^41^,^42^,^43^,^44^, or computational method^38^,^39^,^45^.

The C-B and C-N interface energies per bond are estimated at 0.03 eV and 0.9 eV, respectively. However, since the exact chemical environment during BN island formation is unknown, we introduce the B atom chemical potential *μ*^46^. The zigzag edge energies of BN embedded in Ni(111), and G-BN interface energies with and without the substrate are shown in Fig. 4b and c as a function of *μ*. The C-B energy is clearly lower if the substrate is included, whereas the C-N energy changes only slightly. This is possibly due to the strong interaction with the substrate even though the adsorption energies are small^45^. Moreover, the G-BN interface energies are lower than the energies of BN zigzag edges embedded in Ni(111), especially for C-B, which partly explains why graphene preferentially nucleates at BN edges.

In the shaded region in Fig. 4b,c, the edges of BN embedded in Ni(111) are likely in the N terminated zigzag edge directions. However, after graphene growth the C-N interface has a higher energy than C-B interface, and it is more likely to reconstruct, increasing the number of energetically more favourable C-B bonds. However, the vast majority of interfaces observed in the experiment appear to be stable even at elevated temperatures. Thus, the chemical environment *μ* would have to heavily favour N atoms in order to form stable C-N interfaces. Furthermore, we have shown that the C-B or C-N interfaces do not reconstruct to Klein edges (see supplementary information). We conclude that the interfaces seen in the experiments are most likely C-B interfaces.

## Conclusions

We have developed a new method for synthesizing high quality ZZ oriented G-BN interfaces of more than 150 nm length by sequential deposition of BN and graphene on the strongly interacting Ni(111) surface. BN islands embedded in nickel terraces with atomically sharp ZZ edges serve as seeds for graphene growth. The surrounding nickel terrace seems to play a vital role in this process by stabilising the original BN seed as well as the emerging graphene structure. The size and shape of the graphene domains can be tuned by carefully controlling the preparation conditions. The total length of such interfaces is only limited by the size of the BN seed and atomically sharp interfaces in excess of 150 nm length are demonstrated. A combination of STM and DFT suggests that the interfaces are most likely of ZZ C-B type.

The growth process described here may be readily generalised to nickel foils or epitaxial thin films similar to those used in other G-BN synthesis schemes. Transfer techniques for graphene from nickel foils are well documented in the scientific literature^47^,^48^,^49^. Combining the transfer techniques with our synthesis protocol should enable the manufacture of atomically well-defined G-BN structures of unprecedented quality and their transfer to insulating substrates e.g. for transport experiments.

## Methods

### Experimental

Samples were prepared by sequential CVD growth of BN from borazine (B_3_N_3_H_6_) and graphene from ethylene on a Ni(111) single crystal in ultra-high vacuum (UHV). The crystal was cleaned by repeated cycles of sputtering with neon ions followed by annealing at 1270 K. After the final sputtering cycle and before annealing, the surface was exposed to 30 L of oxygen at room temperature. The oxygen treatment removes any remaining carbon contamination from the crystal surface during annealing^23^. To grow BN, the surface was heated to 1070–1120 K and borazine was introduced into the chamber through a leak valve at pressures in the 10^−9^ to low 10^−8^ mbar region for up to three minutes. After the desired exposure had been reached, the borazine supply was cut and the sample cooled to the graphene growth temperature immediately. Graphene growth was performed at temperatures between 830 K and 850 K by exposing the BN samples to ethylene at pressures in the low 10^−7^ mbar range. To obtain jagged graphene domains, the pressure was raised to the mid 10^−6^ range for a ca. 1 second at the start of the growth interval. After the desired exposure time, the ethylene supply was shut off and samples were annealed at the growth temperature for 15 to 30 minutes to improve the graphene quality. Further details can be found in the supporting information. All STM experiments were carried out in UHV low-temperature STM (Unisoku USM-1300) at *T* = 77 K. The STM images were post-processed using the Gwyddion (http://gwyddion.net/) and WSxM (http://www.nanotec.es) scanning probe microscopy softwares^50^,^51^.

### Numerical Methods

The all-electron FHI-aims package^52^ was used for the first principles calculations. We employed the default tight basis sets for carbon, boron and nitrogen atoms and the default light basis set for nickel atoms. The chosen exchange-correlation energy functional was the generalized gradient approximation as parametrized by Perdew, Burke and Ernzerhof (GGA-PBE)^53^. The self-consistency cycle was considered converged if, among other things, the difference in total energy between consecutive steps was less than 10^−6^ eV. The atomic structures were fully relaxed until the forces present were less than 10^−3^ eV/Å. The Ni(111) substrate was modeled as three layers below h-BN and graphene. The two lowest layers were fixed to bulk positions with lattice constant 2.488 Å. As nickel is a ferromagnetic material, the calculations allowed spin polarization, and the initial spin moments were chosen to closely match the bulk values. Moreover, we enabled the available relativistic treatment, the scaled zero-order regular approximation, in order to treat the heavier nickel atoms in a proper way. Further computational details specific to each model system can be found in the supplementary information.

## Additional Information

**How to cite this article**: Drost, R. *et al.* Synthesis of Extended Atomically Perfect Zigzag Graphene - Boron Nitride Interfaces. *Sci. Rep.* **5**, 16741; doi: 10.1038/srep16741 (2015).

## Supplementary Material

Supplementary Information
