# Supplementary material for: Synthesis of Extended Atomically Perfect Zigzag Graphene - Boron Nitride Interfaces
Source: Sci Rep. 2015 Nov 20;5:16741. doi: 10.1038/srep16741 (PMC4653630; doi:10.1038/srep16741)
Supplement: Supplementary Information [file srep16741-s1.pdf]

Supporting information for: Synthesis of extended atomically perfect zigzag Graphene - Boron Nitride Interfaces

Robert Drost,[†] Shawulienu Kezilebieke,[†] Mikko Ervasti,[‡] Sampsa K. Hämäläinen,[†] Fabian Schulz,[†] Ari Harju,[‡] and Peter Liljeroth[*,†]

Department of Applied Physics, Aalto University School of Science, PO Box 15100, 00076 Aalto, Finland, and COMP Centre of Excellence, Department of Applied Physics, Aalto University School of Science, PO Box 11100, 00076 Aalto, Finland

E-mail: peter.liljeroth@aalto.fi

Sample Preparation

All samples were grown on a (111) terminated nickel single crystal obtained from MaTecK GmbH. The crystal was cleaned in ultra-high vacuum (UHV) by a series of sputtering with neon ions and annealing to temperatures of 1070 K - 1270 K. Prior to the hexagonal boron nitride (BN) growth, the sample was exposed to 30 L of oxygen and heated to 1070 K to remove any remaining surface contamination.

The interaction of the nickel surface with hydrocarbon gases is highly temperature dependent. At relatively low temperatures ($\lesssim$770 K), a surface carbide is formed upon exposure

*To whom correspondence should be addressed

†Department of Applied Physics, Aalto University School of Science, PO Box 15100, 00076 Aalto, Finland

‡COMP Centre of Excellence, Department of Applied Physics, Aalto University School of Science, PO Box 11100, 00076 Aalto, Finland

to hydrocarbons which slowly converts to graphene (G) on a timescale of hours.[S1] The solubility of carbon in the nickel crystal increases substantially above 870 K, leading to the eventual absorption of carbon species on the surface into the bulk.[S2] In between these limits, a narrow temperature window exists where graphene may be grown directly on the surface in a self-limiting fashion. Embedded graphene domains are obtained on clean samples while some residual carbon contamination results in graphene growth on top of the first nickel layer.[S3]

Tight growth temperature constraints do not exists for the growth of BN, but high quality samples are only obtained at temperatures above 1070 K where graphene is not stable on the surface. This leads us to adopting a reverse growth protocol when compared with most other works in the field, beginning with the deposition of BN at high temperatures and finishing the samples by attaching graphene to the BN crystallites at lower temperature. BN was grown by low pressure chemical vapor deposition (CVD) from a borazine ($B_3N_3H_6$, Chemos GmbH) precursor at 1070 K as described in the manuscript.

Figure S1 shows sketched p-T-diagrams for the preparation of pristine BN seeds as well as the attachment of straight or jagged graphene to the seeds.

Additional Structural Information

The local work function of a surface can be measured in STM by performing dI/dV spectroscopy in the field emission regime. The work function of G and BN may be measured on samples containing only one of the two materials and an assignment of materials on the heterolayer may be made or confirmed by comparing the work function measured on different parts of the sample against the references containing only one as shown below. The first peak in either series ($\sim$1.8 V for BN and $\sim$2.3 V for graphene) arise from a quantum well state between the buried Ni(111) surface and the graphene or BN adlayer.[S4,S5]

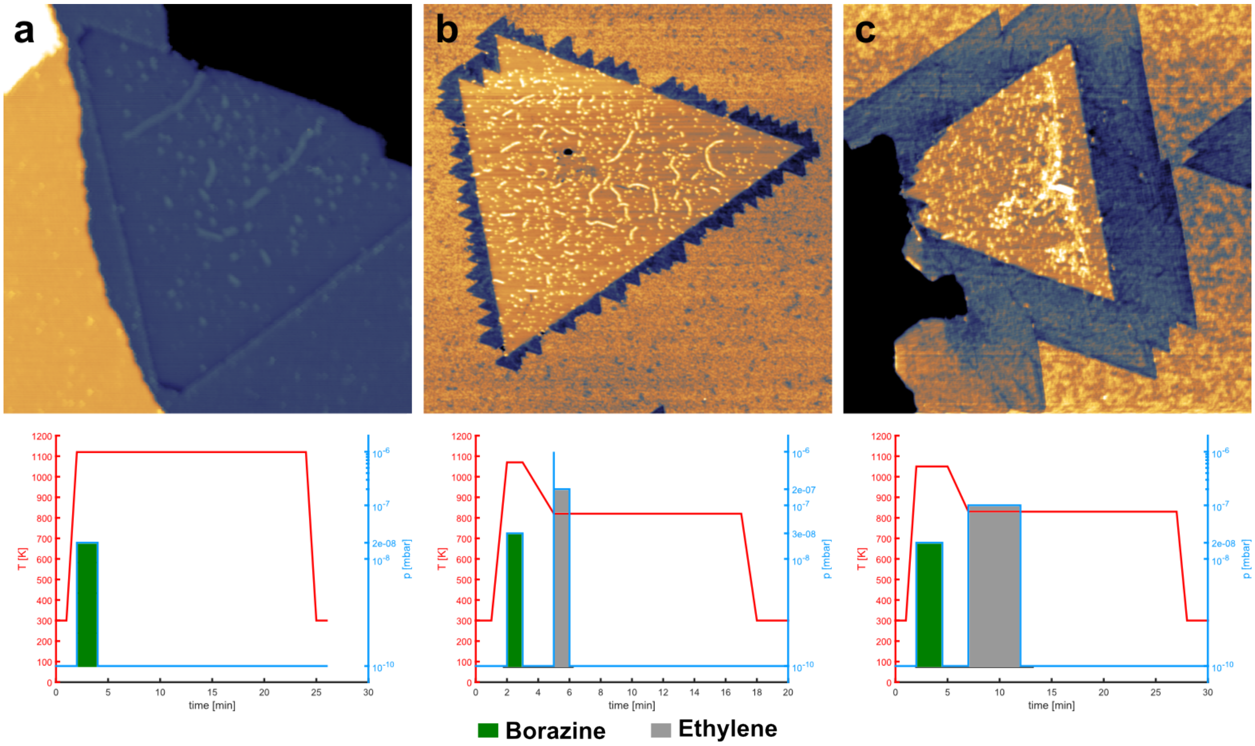

Figure S1: Sketched p-T-diagrams: Preparation conditions for (**a**) pristine BN seeds, (**b**) BN seeds with jagged graphene domains, and (**c**) straight graphene domains

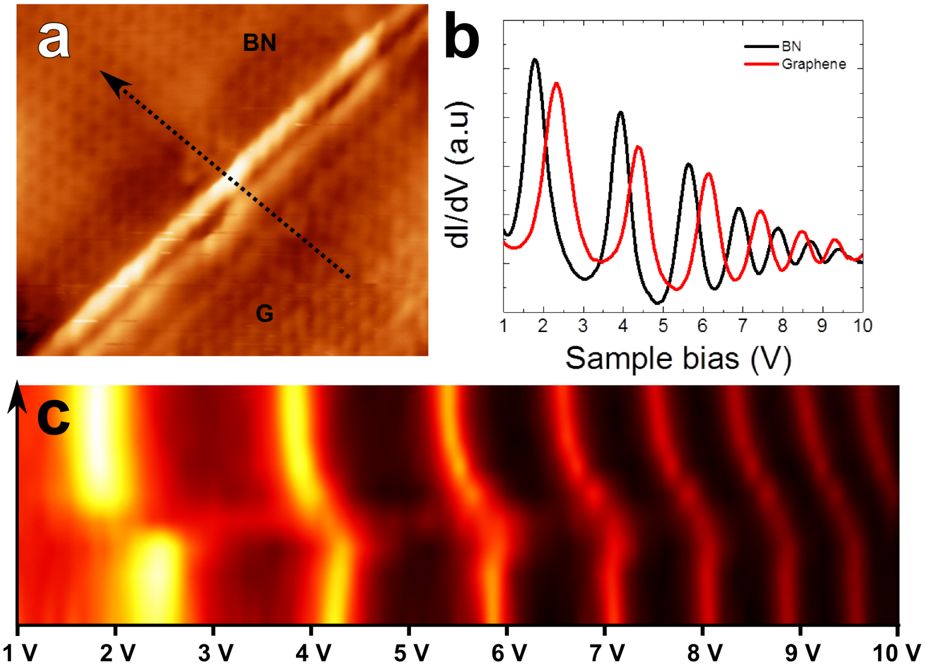

Figure S2: a) Atomically resolved STM image of a G-BN interface. A series of dI/dV spectra in the constant-current mode between 1 V and 10 V are taken along the dotted line. b) Individual sample spectra showcasing the differences between the FERs of G and BN. c) Contour plot of all spectra taken along the dotted line in panel a with an abrupt change of the work function close to the interface region.

Both G and BN grown directly on Ni(111) naturally expose ZZ edges as revealed by atomically resolved STM images close to the G/Ni(111) and BN/Ni(111) interface as shown below.

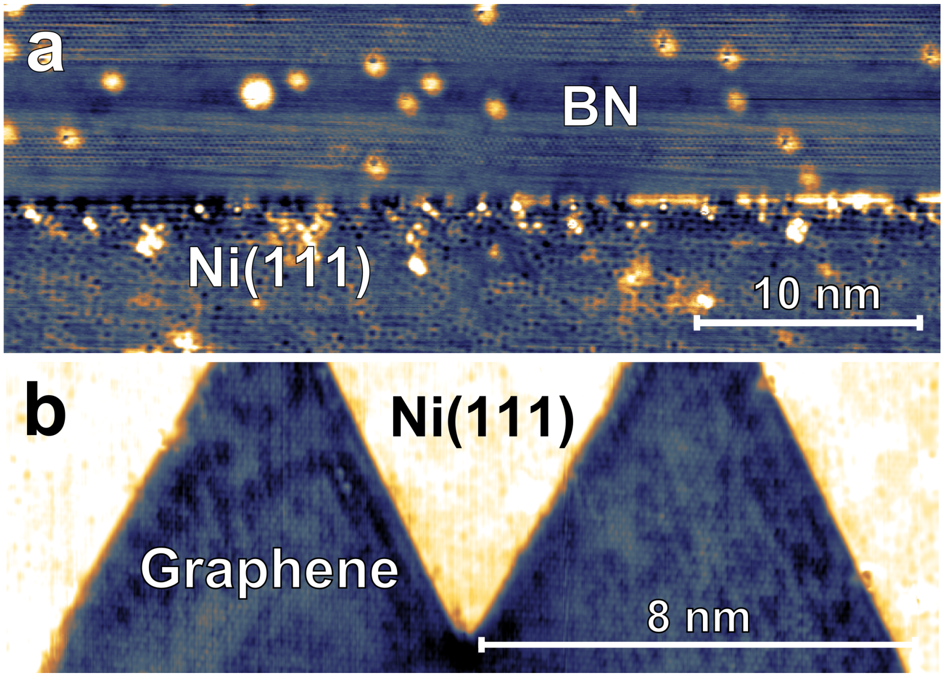

Figure S3: a) STM image near the BN/Ni(111) interface. The BN lattice is resolved in some parts of the image and extrapolation towards the interface reveals it to be ZZ oriented (10 mV, 1 nA). b) Atomically resolved STM image of G triangles attached to a BN edge showing their edges to be ZZ oriented as well (10 mV, 5 nA).

Computational methods and results

We study computationally the edge of hexagonal boron nitride (BN) embedded in Ni(111), and the interface between graphene (G) and h-BN on Ni(111). The experiments show that the interfaces are in the zigzag (ZZ) directions, and therefore we omit the cases with armchair edges in the computations. Moreover, the armchair G/BN interfaces are expected to be unfavourable if either C-B or C-N bonds are energetically more favourable than the other than the other. The main objective is to determine whether the G/BN zigzag interfaces are with C-B or C-N bonds. We also discuss whether the interfaces can reconstruct.

BN embedded in Ni(111)

In order to solve the zigzag edge energies of BN embedded in Ni(111), we devise model systems containing a triangular island of BN, see Figure S4. The supercells are relaxed in a Monkhorst-Pack grid of $8 \times 8 \times 1$ k-points. Such islands with zigzag edges have more B or N-terminated edge sites, associated with the sublattice imbalance. In fact, one requires model systems with more zigzag edge of one type, since the zigzag edge energies cannot be solved in the ribbon geometry alone.[S6]

The interface energy is defined intuitively as the energy of "a system with the interface" minus the energy of "a system without the interface", namely the same number of atoms forming only bulk material. We can write for the edge or interface energy

$$\Gamma = [E - E_s] - [n_{\mathrm{B}}\mu_{\mathrm{B}} + n_{\mathrm{N}}\mu_{\mathrm{N}} + n_{\mathrm{Ni_{fcc}}}\mu_{\mathrm{Ni_{fcc}}}] \,, \qquad (1)$$

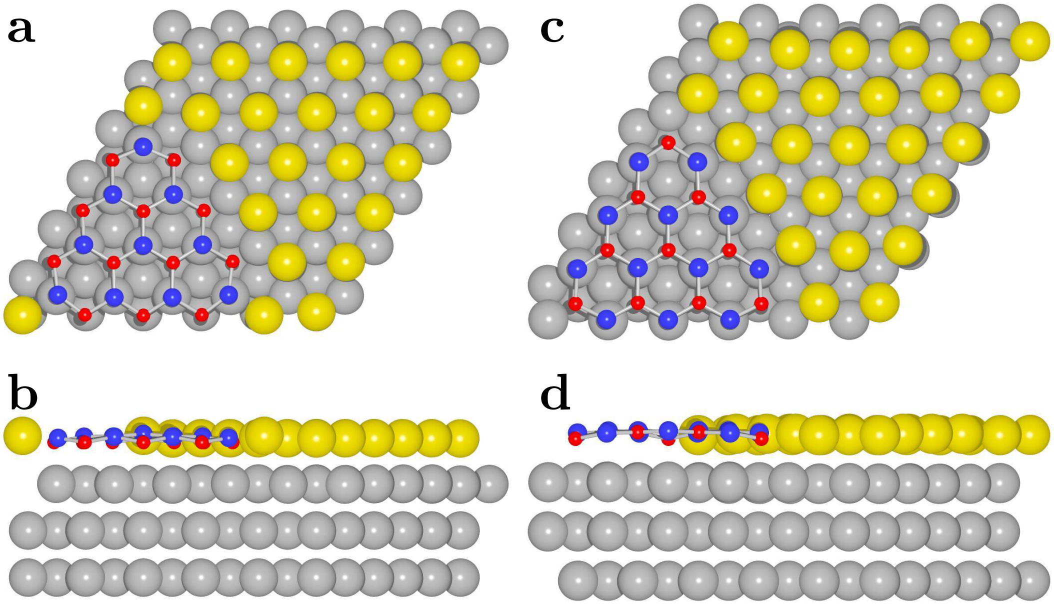

Figure S4: The relaxed computational supercells, viewed from the top and side, containing a triangular zigzag nanoisland of BN embedded in Ni(111) with B-terminated (a-b) and N-terminated (c-d) edges. The red and blue spheres represent boron and nitrogen atoms, and the grey and yellow spheres represent nickel atoms below and at the top layer of the Ni(111) substrate.

where E is the energy of the computational supercell, E_s is the energy of the substrate only, n_i is the number of atoms of type i excluding the substrate, and μ_i is the energy of an atom of type i, forming BN or the top layer of Ni(111), both adsorbed on the substrate. Even if it is not explicitly visible in the notation, the energies μ_i contain the adsorption energy and the formation energy of the bulk material.

A triangular BN nanoisland has a total of $3L$ edge sites at the triangle sides and three additional sites at the corners. Therefore, we can decompose the total edge energies of the two model systems, see Figure S4 and Figure 4 in the main manuscript, as

$$\Gamma_{\triangle} = 3\left(L\gamma_{\mathrm{B}} + \gamma_{\mathrm{N}}\right) \tag{2}$$

$$\Gamma_{\bigtriangledown} = 3\left(\gamma_{\mathrm{B}} + L\gamma_{\mathrm{N}}\right), \tag{3}$$

where $\gamma_{\mathrm{B,N}}$ are the B and N-terminated zigzag edge energies per edge atom. Evaluating the model system total edge energies $\Gamma_{\triangle}$ and $\Gamma_{\bigtriangledown}$ by using Eq. 1, readily allows us to solve for γ_{B} and γ_{N}.

By considering the total edge energy Γ of a system containing both the B and N-terminated triangular nanoislands, and requiring $\Gamma = \Gamma_{\triangle} + \Gamma_{\bigtriangledown}$, it follows that

$$\mu_{\mathrm{B}} + \mu_{\mathrm{N}} = \mu_{\mathrm{BN}}, \tag{4}$$

where μ_{BN} is the energy of a two-atom unit cell of BN on the substrate. The energies or chemical potentials of individual B and N atoms, μ_{B} and μ_{N}, are therefore directly related. Furthermore, to allow any chemical environment preferring either more B or more N atoms in the system, we write

$$\mu_{\mathrm{B,N}} = \mu^0_{\mathrm{B,N}} + \varepsilon \pm \mu, \tag{5}$$

where $\mu^0_{\mathrm{B,N}}$ are the reference energies that we take as the energies of isolated B and N atoms, ε is a constant of $\frac{1}{2}\left(\mu_{\mathrm{BN+s}} - E_s - \mu^0_{\mathrm{B}} - \mu^0_{\mathrm{N}}\right)$ to meet the requirement of Equation (4), and μ

is a free parameter and chemical potential for B atoms, also directly related to the chemical potential of N atoms. If $\mu = 0$, both B and N atoms are at the same potential with respect to their reference energies, and neither is energetically preferred in the system. Otherwise μ controls whether the system is B or N rich with more zigzag edge of the that kind.

The zigzag edge energies of BN embedded in Ni(111) can now be solved

$$\gamma_{\mathrm{B}} = 0.65\ \mathrm{eV} - \frac{1}{3}\mu$$
$$\gamma_{\mathrm{N}} = 1.00\ \mathrm{eV} + \frac{1}{3}\mu. \quad (6)$$

The B-terminated zigzag edges are preferred if $\mu > -0.53$ eV. However, it is difficult to estimate the value of μ in the experiments.

It is well-known that the bare zigzag edge of BN reconstructs to an alternating pentagon-heptagon (57) structure.[S7–S9] The zigzag edge can be stable only if there are for instance hydrogen atoms passivating the dangling bonds. In our case, the BN nanoisland is embedded in the topmost layer of the Ni(111) substrate, and the nearby nickel atoms are expected to bond to the BN edge. Moreover, the lattice structure is not likely broken, excluding 57-type reconstruction, since the nitrogen and boron atoms prefer to reside at the top and fcc sites on the Ni(111) substrate.

Graphene/BN interfaces on Ni(111)

To evaluate the G/BN interface energies, we have devised a computational supercell containing a 22-atom triangular nanoisland of BN embedded in graphene on the Ni(111) substrate. The supercell is relaxed in a Monkhorst-Pack grid of $8 \times 8 \times 1$ k-points, and the relaxed atomic structures are shown in Figure S5. Such systems are already qualitatively similar to what has been measured in the experiments.

As in the case of BN islands embedded in Ni(111), there are more B or N atoms in the model systems, which also translates into having more C atoms occupying the other

sublattice associated with the top or fcc sites. This has to be taken into account when evaluating the interface energies

$$\Gamma = [E - E_s] - \left[n_B\mu_B + n_N\mu_N + n_{\mathrm{C_{top}}}\mu_{\mathrm{C_{top}}} + n_{\mathrm{C_{fcc}}}\mu_{\mathrm{C_{fcc}}}\right]. \quad (7)$$

Again, the energies of individual atoms are assumed to sum up to the energies of bulk BN and graphene on the substrate. We can estimate the carbon atom energies $\mu_{\mathrm{C_{top}}}$ and $\mu_{\mathrm{C_{fcc}}}$ by assuming the following set of equations

$$\mu_{\mathrm{C_{top}}} + \mu_{\mathrm{C_{hcp}}} = \mu_{\mathrm{G_{top\text{-}hcp}}} \quad (8)$$

$$\mu_{\mathrm{C_{top}}} + \mu_{\mathrm{C_{fcc}}} = \mu_{\mathrm{G_{top\text{-}fcc}}} \quad (9)$$

$$\mu_{\mathrm{C_{hcp}}} + \mu_{\mathrm{C_{fcc}}} = \mu_{\mathrm{G_{hcp\text{-}fcc}}}, \quad (10)$$

where in each case the adsorption distances are fixed to the top-fcc value. This ensures that

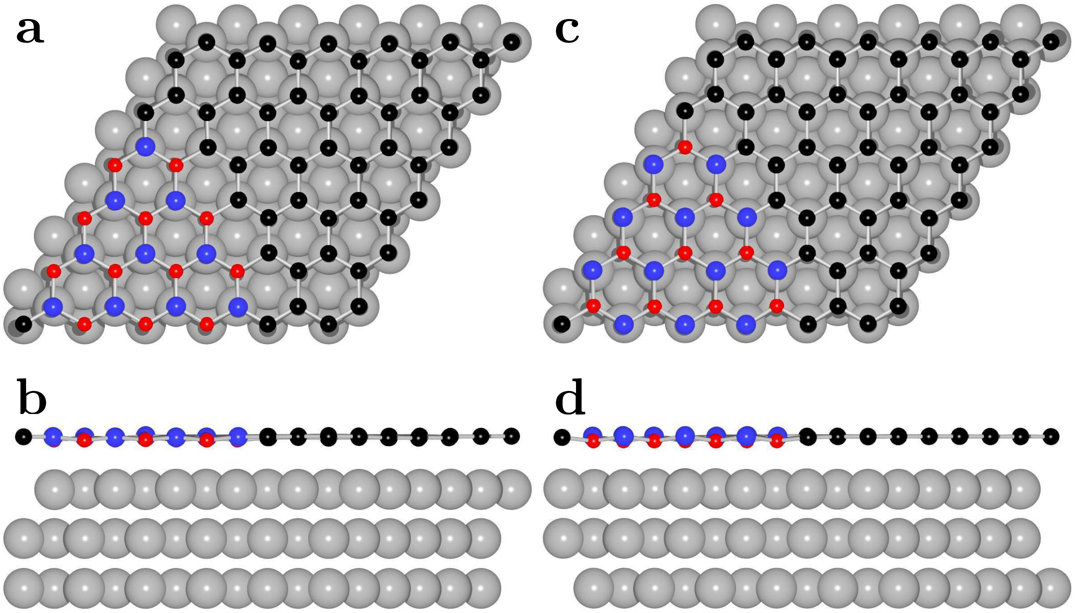

Figure S5: The relaxed computational supercells, viewed from the top and side, containing a triangular zigzag nanoisland of BN embedded in graphene on Ni(111) with C-B (a-b) and C-N (c-d) interfaces at the triangle sides. The red, blue, black and grey spheres represent boron, nitrogen, graphene and nickel atoms, respectively.

the energies of carbon atoms on each site are comparable. The three equations can be solved, and we get that $\mu_{\mathrm{C_{top}}}$ is 0.40 eV lower than $\mu_{\mathrm{C_{fcc}}}$, which clearly implies that the sublattice imbalance of C atoms has to be taken into account.

The zigzag C-B and C-N interface energies per bond can be evaluated in the same manner as in the case of BN embedded in Ni(111). The interface energies are

$$\begin{aligned} \gamma^s_{\text{C-B}} &= 0.03\ \text{eV} - \frac{1}{3}\mu \\ \gamma^s_{\text{C-N}} &= 0.88\ \text{eV} + \frac{1}{3}\mu, \end{aligned} \tag{11}$$

where μ is the chemical potential as defined in Eq. (5). We have also evaluated the interface energies without the substrate, obtaining

$$\begin{aligned} \gamma_{\text{C-B}} &= 0.47\ \text{eV} - \frac{1}{3}\mu \\ \gamma_{\text{C-N}} &= 0.87\ \text{eV} + \frac{1}{3}\mu. \end{aligned} \tag{12}$$

It is expected that the honeycomb lattice at the interface is not broken even if the interface reconstructs in some way. Namely, since the B, N and C atoms prefer the top and fcc sites on the substrate, there would be an associated energy cost to moving the atoms elsewhere, for instance by bond rotation or by formation of 57-type reconstructions. Additionally, there are no dangling bonds at the G/BN interface that would need relaxing. We have therefore restricted our study of the possible interface reconstructions to Klein-type interfaces.

We have relaxed periodic systems with BN ribbons embedded in graphene on Ni(111), see Figure S6a-c. The ribbons have two interfaces that correspond to the edge directions of the two distinct triangular nanoislands. First, we simulated a system with both C-B and C-N zigzag interfaces, see Figure S6a, containing 6 B, 6 N, and 12 C atoms in the computational unit cell that was relaxed using a grid of $4 \times 40 \times 1$ k-points. Moreover, the carbon atom closest to the N-terminated zigzag edge of BN had to fixed to its adsorption distance, since

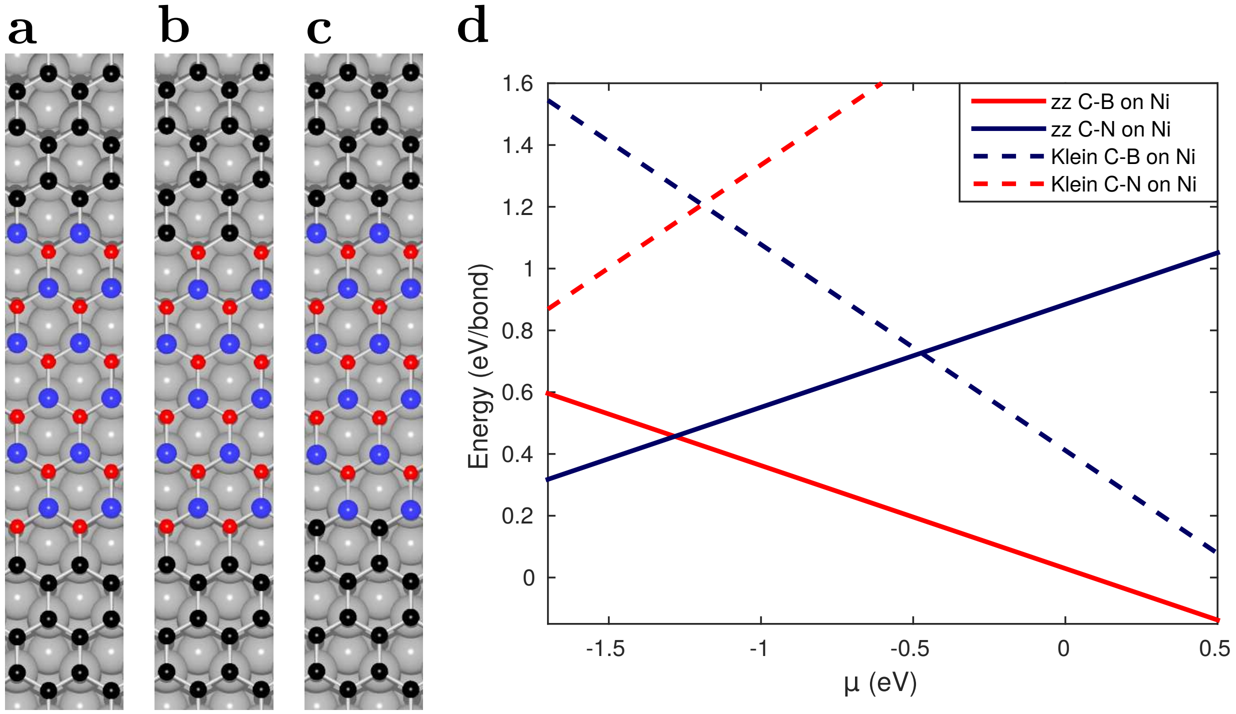

Figure S6: G/BN interfaces in ribbon geometry. (a) Ribbon with zigzag C-N and zigzag C-B interfaces. (b) Ribbon with Klein C-B and zigzag C-B interfaces. (c) Ribbon with zigzag C-N and Klein C-N interfaces. (d) The interface energies as a function of B atom chemical potential μ. The colors represent the distinct edge directions.

otherwise nearby carbon atoms would not stay adsorbed on the substrate. This could be physical, or it can also be an artifact from the PBE exchange-correlation functional. We did not have the same problem with the model systems containing the triangular nanoislands, most likely due to the more complicated geometry where the interface cannot fold as easily. In any case, the sum of the zigzag interface energies is

$$\Gamma = \gamma^{s}_{\text{C-B}} + \gamma^{s}_{\text{C-N}} = 0.85 \text{ eV}, \tag{13}$$

which agrees very well with the energies in Eq. 11 that were obtained using the geometries with triangular nanoislands.

The energies of the Klein interfaces can be readily evaluated by slightly modifying the zigzag interfaces. The relaxed ribbon systems containing Klein interfaces are shown in Figure S6b and S6c. Here again, the carbon atoms next to the nitrogen atoms are fixed to the

graphene adsorption distance. The resulting Klein interface energies are

$$\gamma^s_{\text{Klein(C-B)}} = 0.41 \text{ eV} - \frac{2}{3}\mu$$
$$\gamma^s_{\text{Klein(C-N)}} = 2.0 \text{ eV} + \frac{2}{3}\mu. \tag{14}$$

We have plotted the zigzag and Klein interface energies in Figure S6d. It is clear that the Klein interfaces have much higher energies at reasonable values of μ. Furthermore, in a N-(B-)rich system the BN edges are most likely in the N-(B-)terminated zigzag edge directions, but after graphene growth this direction supports only the C-B (C-N) Klein edge that has more B (N) atoms. Therefore we conclude that the zigzag interfaces are not likely to reconstruct.

References

(S1) Lahiri, J.; Miller, T.; Adamska, L.; Oleynik, I. I.; Batzill, M. Graphene growth on Ni(111) by transformation of a surface carbide. *Nano Lett.* **2010**, *11*, 518–522

(S2) Addou, R.; Dahal, A.; Sutter, P.; Batzill, M. Monolayer graphene growth on Ni(111) by low temperature chemical vapor deposition. *Appl. Phys. Lett.* **2012**, *100*, 021601

(S3) Patera, L. L.; Africh, C.; Weatherup, R. S.; Blume, R.; Bhardwaj, S.; Castellarin-Cudia, C.; Knop-Gericke, A.; Schloegl, R.; Comelli, G.; Hofmann, S.; Cepek, C. In Situ Observations of the Atomistic Mechanisms of Ni Catalyzed Low Temperature Graphene Growth. *ACS Nano* **2013**, *7*, 7901–7912

(S4) Silkin, V.; Zhao, J.; Guinea, F.; Chulkov, E.; Echenique, P.; Petek, H. Image potential states in graphene. *Phys. Rev. B* **2009**, *80*, 121408

(S5) Bose, S.; Silkin, V. M.; Ohmann, R.; Brihuega, I.; Vitali, L.; Michaelis, C. H.; Mallet, P.; Veuillen, J. Y.; Schneider, M. A.; Chulkov, E. V.; Echenique, P. M.; Kern, K. Image potential states as a quantum probe of graphene interfaces. *New J. Phys* **2010**, *12*, 023028

(S6) Liu, Y., Bhowmick, S., Yakobson, B. I., BN white graphene with colorful edges: The energies and morphology. *Nano Lett.* **11**, 3113–3116 (2011).

(S7) Mukherjee, R., Bhowmick, S., Edge stabilities of hexagonal boron nitride nanoribbons: A first-principles study. *J. Chem. Theory Comput.* **7**, 720-724 (2011).

(S8) Hu, T., Han, Y., Jinming Dong, Edge reconstructions of hexagonal boron nitride nanoribbons: A first-principles study. *Physica E* **54**, 191-196 (2013).

(S9) Zhao, R., Gao, J., Liu, Z., Ding, F., The reconstructed edges of the hexagonal BN. *Nanoscale* **7**, 9723–9730 (2015).
